# The use of *Ephedra* herbs in the treatment of COVID-19 

**DOI:** 10.22038/AJP.2022.21607

**Published:** 2023

**Authors:** Amin Sadeghi Dousari, Marzieh Karimian Amroabadi, Zahra Soofi Neyestani, Majid Taati Moghadam, Naghmeh Satarzadeh

**Affiliations:** 1 *Department of Microbiology, School of Medicine, Jiroft University of Medical Sciences, Jiroft, Iran*; 2 *Department of Biology, Science and Research Branch, Islamic Azad University, Tehran, Iran*; 3 *Department of Psychology, Faculty of Literature and Human Science, University of Malayer, Malayer, Iran*; 4 *Department of Microbiology, School of Medicine, Iran University of Medical Sciences, Tehran, Iran*; 5 *Student Research Committee, Faculty of Pharmacy, Kerman University of Medical Sciences, Kerman, Iran*; 6 *Department of Pharmaceutical Biotechnology, Faculty of Pharmacy, Kerman University of Medical Sciences, Kerman, Iran*

**Keywords:** Ephedra, Traditional medicines, COVID-19, Respiratory infections

## Abstract

**Objective:**

*Ephedra* herbs are the only extant genus in its family, Ephedraceae, and order, Ephedrales. It has been prescribed in traditional medicine for improving headaches and respiratory infections. On the other hand, because the coronavirus disease 2019 (COVID-19) causes respiratory problems and COVID-19 pandemic is the most widespread outbreak that has affected humanity in the last century, the current review aims using literature search to investigate the effects of the *Ephedra* herbs compounds on COVID-19 to supply a reference for its clinical application in the inhibition and remedy of COVID-19.

**Materials and Methods:**

This review was performed using articles published in various databases, including Web of Science, PubMed, Scopus, and Google Scholar, without a time limit. For this paper, the following keywords were used: "*Ephedra*", "coronavirus disease 2019", "COVID-19", "Severe acute respiratory syndrome coronavirus 2" or "SARS CoV 2".

**Results:**

The results of this review show that the *Ephedra* herbs have effectiveness on COVID-19 and its compounds can bind to angiotensin-converting enzyme 2 (ACE2) with a high affinity and act as a blocker and prevent the binding of the virus.

**Conclusion:**

Some plants used in traditional medicine, including the *Ephedra* herbs, with their active compounds, can be considered a candidate with high potential for the control and prevention of COVID-19.

## Introduction

The coronavirus was first identified by Bynoe and Tyrrell in 1965 from the respiratory tract sample of a patient with a common cold after culturing in the human embryonic trachea (Dousari et al., 2020; Kahn et al., 2005). The coronavirus is a member of the Coronaviridae family and contains the RNA genome (Gorbalenya et al., 2020). Coronaviruses such as HCoV-229E, HCoV-HKU1, HCoV-NL63, and HCoV-OC43 have been presented as viruses with mild virulent for human (WHO, 2020). In 2019, a severe respiratory infection was reported in Wuhan which could involve lung cells with the SARSCoV2 virus and subsequently the disease was announced as a pandemic by the World Health Organization (WHO) (Moghadam et al., 2021; Shakibnia et al., 2021; Chan et al., 2020; Jin et al., 2020). Clinical findings indicate that the symptoms of COVID-19 disease vary from respiratory problems to septic shock (Sahin et al., 2020). Moreover, the disease has a high mortality rate (Taati Moghadam et al., 2021). Manifestations of COVID-19 become visible approximately five days after contamination and last a minimum of 41 days and a maximum to the end of life with a norm of 14 days (Li et al., 2020). The main symptoms of COVID-19 disease are myalgia or fatigue, cough, and fever (Zu et al., 2019). Although this is mild in most people, some patients, particularly those with underlying diseases, may have arrhythmia, respiratory failure, kidney failure, shock, cardiovascular damage, or liver failure (Alimohamadi et al., 2020; Atique et al., 2020; Guo et al., 2020). 

It is a fact that the COVID-19 pandemic is the most widespread that has plagued humanity in the last century. Hence, researchers in different areas of the world are seeking to discover a potential therapy for COVID-19 (Besharati et al., 2022; Hashem-Dabaghian et al., 2022; Arokiaraj et al., 2020). The idea of utilizing herbs for therapeutic goals has existed since the beginning of recorded human history and has been the source of a lot of recent medicine (Barkat et al., 2021; DiPietro et al., 2021). Health officials of many countries, especially China and India, recommend the use of traditional herbal remedies as an alternative to preventative measures to help those with mild to moderate respiratory infections, so, using herbs for the diseases are not a new idea. Traditional science is essential function in prescribing herbs as a treatment and discovering influential medicines to address improving health problems (Sadeghi Dosari et al., 2016). Today, after tens of centuries, medicinal plants such as holy basil, black pepper, garlic licorice, cloves, turmeric, caraway, cardamom, cinnamon, ephedra, and ginger are still as a preventative measure for some diseases such as fever, common cold, and influenza pneumonia (Arokiaraj et al., 2020). Furthermore, one of the first drugs suggested to treat COVID-19 was hydroxychloroquine derived from the herb species Cinchona ( DiPietro and Mondie, 2021; Liu et al., 2015).

*Ephedra* herbs belong to the family Ephedraceae. The medicinal plants have about 50 species (Elhadef et al., 2020; Benabderrahim et al., 2019; Wang et al., 2006) (the important species of *Ephedra* are listed in Table 1). The shrubs of this plant, which reach a height of about one meter, grow in semi-arid and desert circumstances in both hemispheres on all continents ( Yang et al., 2018; Caveney et al., 2001). The *Ephedra* extract includes several alkaloids, such as the primary active constituent, ephedrine, a small amount of pseudoephedrine, methylephedrine, phenylpropanolamine, norpseudoephedrine, and methylpseudoephedrine (Dosari et al., 2022; Kalman et al., 2003; Gurley et al., 199). Besides, the herb consists of phenolic compounds including aromatic compounds, lignans, flavonoids, and proanthocyanidins (González-Juárez et al., 2020). *Ephedra sinica *is one of the ancient medicinal plants in traditional Chinese therapy which is known as “Ma Huang” ( Eng et al., 2019; Sõukand et al., 2015). It has been used for over 5000 years as an anti-asthmatic and stimulant and for treating allergies, bronchial asthma, cough, cold, flu, headache, edema, and fever (Elhadef et al., 2020; Thakur et al., 2018). This review discusses the use of *Ephedra* and its compounds in treating COVID-19 disease.

## Materials and Methods

This review was performed on articles indexed in various databases, including Web of Science, PubMed, Scopus, and Google Scholar, without a time limit. For this paper, the following keywords were used: "*Ephedra*", "coronavirus disease 2019", "COVID-19", "Severe acute respiratory syndrome coronavirus 2" or "SARS CoV 2". All articles containing the use of *Ephedra* herbs and their compounds in the treatment of COVID-19 were assessed in this review. The search for articles was limited to the English language and original full-text articles.

## Results

**Can the compounds in** ***Ephedra*** **herbs be a tool to prevent or treat COVID-19?**

Traditional Chinese medicines (TCM) can play an influential duty in the remedy and prevention of COVID-19 (Li et al., 2021). In the course of the pandemic, the health committees and administrations of TCM of all states of China have prepared Chinese medicine therapy and prevention schedules for this pandemic according to the general symptoms of the sick people. A study has examined prescriptions for prevention and treatment of COVID-19 and discovered that ephedra- bitter almond was one of the main commonly used medicines (Cheng et al., 2020). For example, Qingfei Paidu decoction contains the couplet medicines of ephedra-bitter almond, so it is recommended in the therapy of all steps of COVID-19 via Treatment Protocol for Novel Coronavirus Pneumonia and National Health Committee and Local Diagnosis (Zhao et al., 2020). The success rate of this decoction for COVID-19 has been announced to be more than 90% (Ren et al., 2020). 

When Qingfei Paidu decoction (Qingfei Paidu medicine consists of 21 compounds (197.5 grams), of which 9 grams (4.56%) are related to the *Ephedra* plant) was utilized as a complementary therapy to western medicine, it could improve the regression of lung inflammation and symptoms and demonstrate a tendency to decrease the level of multi-organ impairment (Xin et al., 2020). Furthermore, statistics exhibited that *Ephedra* and *Glycyrrhiza* are frequently used in the therapy of COVID-19 (Wang et al., 2020; Zhou et al., 2020). *Ephedra* demonstrated anti-inflammatory, antioxidant, antibacterial, antiviral, and diuretic effects (Zhang et al., 2018). Also, it is widely employed in respiratory illnesses, for example, colds, influenza, and asthma and could remove some symptoms such as nasal congestion, fever, cough, and headache (González-Juárez et al., 2020). The Ephedra-Glycyrrhiza duplet medicine was documented in Zhang Zhongjing in the Golden Chamber Synopsis which has been frequently prescribed for the remedy of bronchial asthma and colds (Wang et al., 2012). Both *Ephedra* and *Glycyrrhiza* have exhibited anti-inflammatory and immunomodulatory effects (Nomura et al., 2019; Panaampon et al., 2019; Wei et al., 2019; Qamar et al., 2012). Nevertheless, there was no related studies on the mode of action of this duplet medicine in the therapy of COVID-19.

Li et al. (2021) examined the chemical component and pharmacological mode of action of the *Ephedra-glycyrrhiza* drug against COVID-19. They have identified 112 active components from *Ephedra-Glycyrrhiza* through the network pharmacology method that 23 and 92 active components belonged to *Ephedra* and *Glycyrrhiza*, respectively. This drug pair enrichment analysis showed that they might participate in the cyclic adenosine monophosphate (cAMP), the Janus kinase-signal activator and transducer of transcription (JAK-STAT), and chemokine signaling pathways, and the phosphatidylinositol 3‑kinase-protein kinase B (PI3K-Akt) which has a significance relationship with respiratory tract, blood circulation, digestive and nervous system-related illnesses. Mapping relation examination between COVID-19 and *Ephedra-Glycyrrhiza* exhibited that the key markers were tumor necrosis factor-α (TNF-α), interleukin-2 (IL-2), albumin (ALB), FOS proto-oncogene, and prostaglandin- endoperoxide synthase 2 (PTGS2). The network between the three viral indicators such as S protein, ACE2, and Mpro were associated with covid-19 and in the 112 active compounds showed, 110 active compounds (110/112) could bind to ACE2, all 112 active components affected Mpro, and only 24 of 112 active components could inhibit S protein. The docking results of these 112 active components with S protein, ACE2, and Mpro showed that xambioona has the highest affinity but the lowest binding energy with ACE2, licorice glycoside E had the highest affinity but the lowest binding energy with Mpro and S protein, and gancaonin H showed the lowest affinities but the highest binding energy with Mpro. Therefore, these results exhibited that active components of the pair of *Ephedra-Glycyrrhiza* effectively bind COVID-19 targets such as the main protease ACE2, Mpro, and S protein.

Finally, it is suggested that ephedra-glycyrrhiza might be active on COVID-19 via numerus targets and ways based on molecular dynamics methods, molecular docking, and network pharmacological analysis *in silico*. Although further investigations are required to verify the efficacy of *Ephedra* and *Glycyrrhiza* in the treatment of COVID-19, according to available findings both *Ephedra* and *Glycyrrhiza* showed therapeutic effects against COVID-19 (Li et al., 2021). Many studies have displayed that some TCM prescriptions such as Jinhua Qinggan granule (Liu et al., 2020), Lianhuaqingwen capsule/granule ( Xiao et al., 2020; Hu et al., 2019), Huashi Baidu granule (Sõukand et al., 2015) and Qingfei Paidu decoction (Lee et al., 2021) which include *Ephedra sinica* could successfully prevent the fatal deterioration and reduce the symptoms of COVID-19. 

**Table 1:** Some important species of *Ephedra* plant

Reference	Therapeutic effects	Species of *Ephedra* plant
** (Mei et al., 2021)**	It disrupts ACE2-RBD interaction and prohibits the entrance of pseudoviruses.	*Ephedra sinica*
**(Li et al., 2022)**	MSGSD (including *Ephedra intermedia, Prunus armeniaca, Glycyrrhiza uralensis Fisch., *and* Gypsum fibrosum*) demonstrated a protective impact on RSV-exacerbated asthma and a decrease of Th2 cytokines and neurogenic inflammatory intermediates.	*Ephedra intermedia*
**(Chaitanya et al., 2014)**	Bronchodilatory, antihistamine and anti-cholinergic effects	*Ephedra gerardiana *
**(Zhu et al., 2022)**	Alkaloids of *Ephedra equisetina* demonstrated considerable anti-asthmatic effects.	*Ephedra equisetina*
**(Al-Snafi et al., 2017)**	It was employed in traditional Chinese Pharmacopoeia for the treatment asthma, colds, chills, cough, and allergies.	*Ephedra alata*
**(Askew et al., 2017)**	It was reported in Mexican medicinal plants as a treatment for pleurisy (inflammation of the tissue between the ribcage and lungs) and as an adjunctive treatment for pneumonia.	*Ephedra pedunculata*
**(González-Juárez et al., 2020)**	It was reported to help to treat pneumonia, kidney failure, and venereal illnesses.	*Ephedra aspera*
**(Basit et al., 2021)**	It was utilized to remedy asthma, flu, and nasal congestion.	*Ephedra ciliata*

Mei et al. (2021) have examined the active compounds of *Ephedra sinica* extracts that interrupt the interaction between severe acute respiratory syndrome coronavirus-2-receptor-binding domain (SARS-CoV-2-RBD) and ACE2. There were three active components including 4-hydroxyquinoline-2-carboxylic acid, 4, 6 dihydroxyquinoline-2-carboxylic acid, and 4-hydroxy 6-methoxyquinoline-2-carboxylic acid in extracts of *Ephedra sinica* that could effectively prevent ACE2-RBD interaction（IC50=0.07 μM, 0.58 μM, and 0.15 μM respectively). On the other hand, the main active component in *Ephedra sinica* with potential therapeutic agent for COVID-19 was quinoline-2-carboxylic acids.

In another study, Lv et al. (2021) screened and evaluated anti-SARS-CoV-2 compounds of *Ephedra sinica* via ACE2/cell membrane chromatography-high-performance liquid chromatography- ion trap -time of flight-mass spectrometry (ACE2/CMC-HPLC-IT-TOF-MS) approach. In the study, an ACE2/CMC bio affinity chromatographic model was created, and then an ACE2/CMC HPLC-IT-TOF-MS method was designed to screen and identify the active components of *Ephedra sinica* extract. Surface plasmon resonance (SPR) and molecular docking assays were carried for evaluating the binding characteristics. Also, CCK-8 staining was used for evaluating the level of toxicity of the screened components, and SARS-CoV-2 pseudovirus which exhibits the impact of viropexis on the examined components in ACE2^h^ cells.The compounds including ephedrine (EP), pseudoephedrine (PEP), and methylphedrine (MEP) were separated and identified from *Ephedra sinica*.

**Table 2:** A summary of some of the most important studies that show the effect of different species of *Ephedra* plants on COVID-19

**Reference**	**Result**	**Year**	**Author name**
(Cheng et al., 2020)	The *ephedra*-bitter almond was one of the main commonly used medicines for prevention and treatment of COVID-19.	2020	Cheng et al.,
(Zhao et al., 2020)	The *ephedra*-bitter almond was recommended in the therapy of all steps of COVID-19.	2020	Zhao et al.,
(Xin et al., 2020)	Qingfei Paidu decoction containing Ephedra could improve the regression of lung inflammation and symptoms	2020	Xin et al.,
(Liu et al., 2020)	*Ephedra sinica* could successfully prevent the fatal deterioration and reduce the symptoms of COVID-19	2020	Liu et al.,
(Gao et al., 2020)	Several main components of *ephedra*-bitter almond had a high attaching potential to 3CL and ACE2 as well as provided novel drug development for COVID-19.	2020	Gao et al.,
(Li et al., 2021)	Active components from the pair of *Ephedra-Glycyrrhiza* effectively bound to COVID-19 targets such as the main protease ACE2, Mpro, and S protein, so *Ephedra* and *Glycyrrhiza* were showed therapeutic effects in cope with COVID-19	2021	Li et al.,
(Lee et al., 2021)	Traditional Chinese herbal medicine formulae containing *Ephedra sinensis* showed excellent relief of lung congestion and diarrhea, two characteristics of COVID-19 infection.	2021	Lee et al.,
(Mei et al., 2021)	There was different active component, but the main active component in *Ephedra sinica* with potential therapeutic agent for COVID-19 was quinoline-2-carboxylic acids	2021	Mei et al.,
(Lv et al., 2021)	The active compounds of *Ephedra sinica* had ACE2-binding properties which acted as blockers for preventing SARS-CoV-2 spike pseudovirus	2021	Lv et al.,

Binding assays revealed these three components could attach to ACE2, in a specific way too many amino acid residues, identical to the way SARS-CoV-2 binds to ACE2. Furthermore, these components, specifically EP, could prevent SARS-CoV-2 spike pseudovirus entry to ACE2^h^ cells and can decrease the pseudovirus entry rate in the pseudovirus model. In general, MEP, PEP, and EP of *Ephedra sinica* were acted as blockers for preventing SARS-CoV-2 spike pseudovirus.

Gao et al. (2020) evaluated the main compounds and the mode of action of *Ephedra*-bitter almond for therapy and prevention of COVID-19 according to network pharmacology that couplet medicine played an overall regulatory anti-COVID-19 function via the multi-component-target-pathway patterns. Also, several main components of *Ephedra*-bitter almond, including β-sitosterol, estrone, and stigmasterol, had a high attaching potential to 3CL and ACE2 as shown by molecular docking simulation. As a result, *Ephedra*-bitter almond showed as a novel drug for COVID-19. Nevertheless, this study is a prospective analysis according to data mining, and the results need to be explained with precaution. Table 2 shows a summary of some of the most important studies which exhibited the effect of different species of *Ephedra* plant on COVID-19.

## Discussion

According to the results of various articles were examined in this study, COVID-19 known as a contagious respiratory disease induced by severe acute respiratory syndrome coronavirus 2 (SARS-CoV-2). Presently, there is no approved drug for treating the illness. The *Ephedra* plants are a pharmacological herb that can be utilized for the remedy of respiratory infections. The herb extract contains several active components with therapeutic effects such as ephedrine, pseudoephedrine, methylephedrine, phenylpropanolamine,norpseudoephedrine, methylpseudoephedrine, aromatic compounds, lignans, flavonoids, and proanthocyanidins. The results of this review show that the *Ephedra* herbs compounds can bind to ACE2 with a high affinity, act as a blocker and prevent the binding of the virus. Although this review is the beginning of a comprehensive review of the effect of *Ephedra* on COVID-19, further studies are needed to determine the clinical efficacy of *Ephedra* in order to investigate its use as a definitive treatment, especially for new variants of this infamous virus.

## Conflicts of interest

The authors have declared that there is no conflict of interest.
